# A Fiji process for quantifying fluorescent puncta in linear cellular structures

**DOI:** 10.17912/micropub.biology.001003

**Published:** 2023-12-14

**Authors:** Heino Hulsey-Vincent, Neriah Alvinez, Samuel Witus, Jennifer R. Kowalski, Caroline Dahlberg

**Affiliations:** 1 Biology, Western Washington University; 2 0009-0006-4704-2314, Western Washington University, Bellingham, Washington, United States; 3 Fred Hutch Cancer Center, Seattle, Washington, United States; 4 Western Washington University, Bellingham, Washington, United States; 5 University of California, Berkeley, Berkeley, California, United States; 6 Biological Sciences, Butler University, Indianapolis, Indiana, United States

## Abstract

Understanding the cell biology of protein trafficking and homeostasis requires reproducible methods for identifying and quantifying proteins within cells or cellular structures. Imaging protocols for measuring punctate protein accumulation in linear structures, for example the neurites of *C. elegans,* have relied on proprietary software for a full range of analysis capabilities. Here we describe a set of macros written for the NIH-supported imaging software ImageJ or Fiji (Fiji is Just ImageJ) that reliably identify protein puncta so that they can be analyzed with respect to intensity, density, and width at half-maximum intensity (Full-Width, Half-Maximum, FWHM). We provide an explanation of the workflow, data outputs, and limitations of the Fiji macro. As part of this integration, we also provide two independent data sets with side-by-side analyses using the proprietary IgorPro software and the Fiji macro (Hulsey-Vincent, et al. A, B., 2023 submitted). The Fiji macro is an important new tool because it provides robust, reproducible data analysis in a free, open-source format.

**Figure 1. Identification and analysis of puncta using Fiji.**
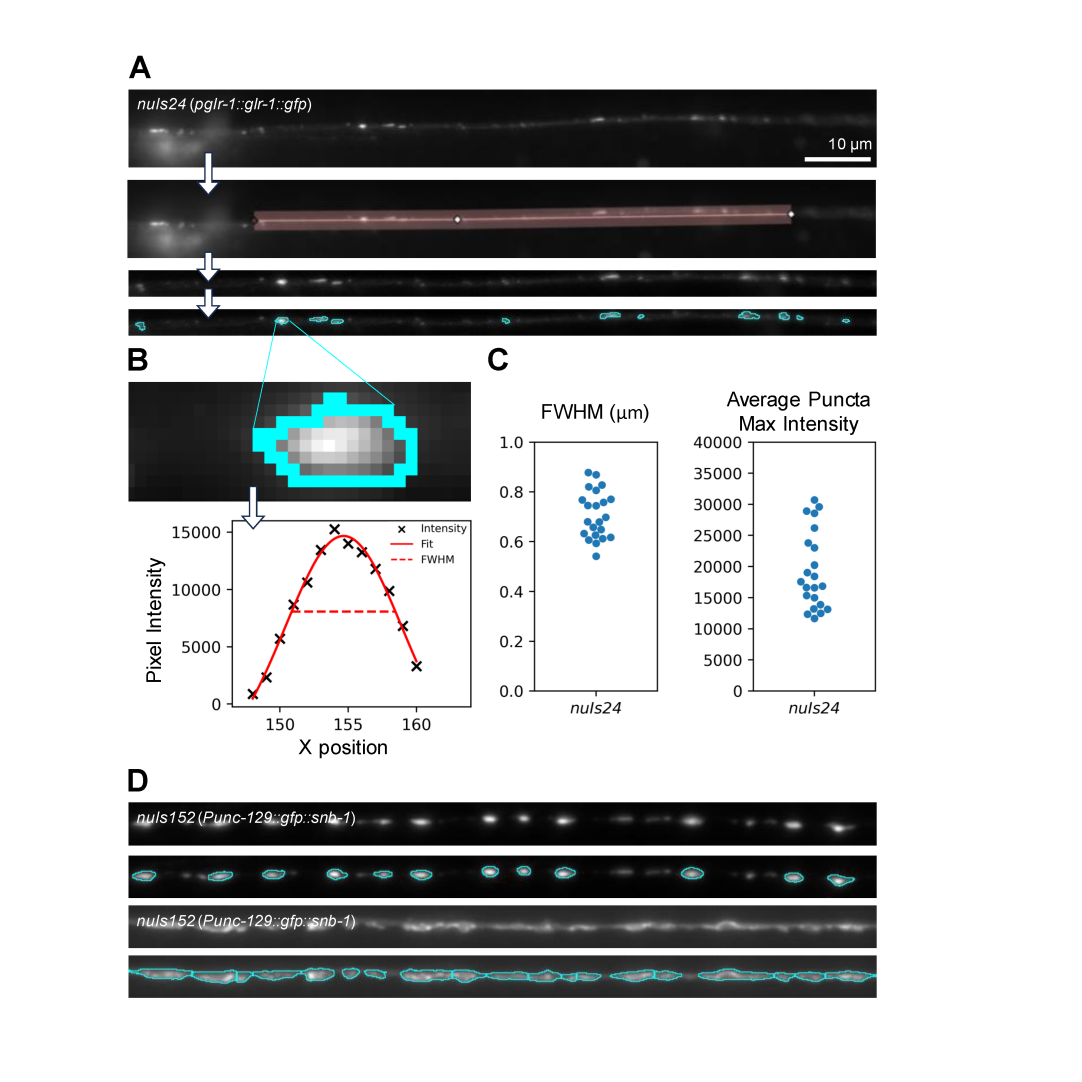
**A.** The Fiji macro straightens a user-defined line-scan to identify fluorescent puncta based on parameters that can be used across datasets. **B.** An intensity profile of the punctum outlined in cyan. Puncta that are defined using the Fiji macro are analyzed so that data about intensity (X) and width (Full-Width at Half-Maximal intensity, (FWHM), dashed line) can be recorded and output as a CSV file. **C.** Example data from ventral nerve cords in animals expressing GLR-1::GFP under the control of the *glr-1* promoter ( *nuIs24* ), N = 22. **D.** Comparison of puncta identification from a sample with distinct puncta (top two panels) and a sample with puncta that are less distinct from the nerve cord (bottom two panels). Images are from dorsal nerve cords in animals expressing GFP::SNB-1 under the control of the *unc-129* promoter ( *nuIs152* ).

## Description

Protein accumulation in cellular structures that include synapses, nuclei, and protein aggregates can be quantified using software or via counting by hand. For example, the localization of synaptic and non-synaptic proteins as well as trafficking intermediates has been analyzed using the Igor software package (Burbea et al., 2002; Dahlberg and Juo, 2014; Dittman and Kaplan, 2006; Kreyden, et al., 2020; Kowalski et al., 2011; Kowalski et al., 2014; McGehee et al., 2015; Sieburth et al., 2005; Ch'ng et al, 2008), MatLab (Kim, et al., 2008) , and using Fiji’s (Fiji Is Just ImageJ) area-under-the-curve or thresholding (Topalidou et al., 2016; Rose et al., 2022; Koushika et al., 2004) . However, Igor, MatLab, and some Fiji approaches for puncta analysis have drawbacks because of cost, reliance on coding skills, and potential loss of important aspects of data for analysis.

Puncta analysis is an important aspect of understanding protein accumulation and dynamics, and we hoped to address some of the problems with current software analysis tools for analyzing puncta in the neurites of *C. elegans* neurons. While the Igor and MatLab software packages are powerful, they are costly and require coding skills. Igor users may require assistance from program creators to adjust analysis parameters to specific microscopes and details of data (for example statistics for individual puncta) can be difficult or impossible to pull out of a larger analysis provided by the Igor software. While some quantification of puncta number or intensity is possible without using Igor, not all parameters are easily available without it. Fiji is equipped with histogram tools and thresholding plugins that can be used to quantify area under a curve (i.e., total fluorescence of a particular line segment) or the area of individual or well-defined puncta (i.e., thresholding to identify puncta vs background). However, neither of the Fiji methods provides the full suite of analysis that is available through Igor. Thus, researchers without access to Igor or MatLab and the coding skills required to modify parameters or connect the programs to different imaging instruments are at a disadvantage with regard to fully analyzing their data.

To democratize access to such puncta analysis methods, as well as to standardize quantification easily across microscope types, we developed a protocol for the ImageJ platform Fiji (Schindelin et al., 2012) . This new protocol reliably identifies puncta across collections of images and can provide data on punctal intensity, width (FWHM), and density (puncta per unit of linear measurement). We have validated this software on animals expressing GLR-1::GFP (Hulsey-Vincent, et al. *B* , 2023) and GFP::SNB-1 (Hulsey-Vincent, et al. *A* , 2023).

The Fiji protocol can be used on TIF images and requires some user input (GitHub ReadMe, https://github.com/heinohv/puncta_analysis ). Users define the width of a linear segment to analyze and provide parameters that ensure that puncta are correctly identified ( Figure 1A ). Once parameters have been set for a given type of puncta reporter (for example, the SNB-1 and GLR-1 GFP fusions described in Hulsey-Vincent, et al. *A, B* , 2023), they can be utilized across different experimental conditions (mutant backgrounds, environmental stimuli, drug treatments, etc.). Moreover, once parameters are set, data analysis on linescans can remain objective and does not require each image (or punctum) to be individually processed by a researcher. Puncta values for intensity and width are computed via the FIJI macro ( Figure 1B ). The output file from analysis reports the data for each punctum, which can be combined *by image* for information *per animal.* The analysis reports also include information on puncta density (Hulsey-Vincent, et al. *A* , *B* 2023). We find that representation of these data can be more illustrative of the biological phenomena compared to bar graphs that have been previously used for the Igor software package ( Figure 1C ). At this time, unlike the Igor program, the FiJi macro does not provide cord fluorescence intensity values (Dreier et al., 2005; Ch'ng et al, 2008). However, because the biological meaning of the cord fluorescence values remains unclear, we do not view this as a major shortcoming of the Fiji macro.

We did encounter a data set for which the Fiji protocol could not identify puncta from a subset of the images ( Figure 1D ). In this case, it appears that when animals have broadly different punctal patterns (for example, a bimodal set of images, some of which have very small puncta with a similar fluorescence intensity to the background linescan), a single set of parameters may not reliably analyze all images. Further examples could be useful in determining whether the disparity in punctum identification can provide, in itself, a binary measure of image quality or biological phenotype in such cases. Overall, the Fiji macro provides an user-friendly, adaptable, and accessible alternative for identification, analysis, and quantification of linear arrays of fluorescent puncta, in this case the ventral and dorsal nerve cords of *C. elegans* ( Figure 1A, Figure 1D, top, Hulsey-Vincent, et al. B, 2023; Hulsey-Vincent, et al. *A* , 2023).

While our data show the efficacy of this software on neural puncta in *C. elegans* , we anticipate that other models of protein accumulation could benefit from this software. For example, analysis of polyQ protein aggregates in *C. elegans* could be streamlined using this macro with Fiji. It is also possible that other linear structures (cultured neural processes, fungal hyphae, or filamentous algae) would be amenable to this tool. We also hope that this accessible software package can be implemented by laboratories at Primarily Undergraduate Institutions (PUIs) or others for whom expensive proprietary software might otherwise be a barrier to data analysis and publication.

## Methods

***Strains and Strain Maintenance***

Strains were grown at 20°C on plates containing nematode growth medium (NGM) agar spotted with OP50 *Escherichia coli* (Brenner et al. 1974). The *C. elegans* strains used in this study were *nuIs24* (P *glr-1::GLR-1::GFP* ) and *nuIs152 (Punc-129::GFP::SNB-1)* that were immobilized in 30mg/mL 2,3-butanedione monoxime (BDM) in M9 and mounted on slides made of 2% agarose pads. For *nuIs24* , the ventral nerve cords of L4 hermaphrodites were imaged using a Leica iDM6000 using a 60X HC Pl Apo NA 1.4 oil immersion objective. 10 micron z-stacks were assembled as max-projection images and analyzed using the Fiji macro (described below). For *nuIs152* , images were acquired as in (Hulsey-Vincent, et al., A 2023) using the Leica DMLB widefield microscope.

**Analysis parameters**

The parameters for analysis are described below.

For *nuIs24* data we used a minimum puncta size = 0.2, sigma = 1, radius = 6, method = Phansalkar. We discarded points where the full width half max was greater than the ROI width (28 discarded and 1601 kept).

For *nuIs152* data we used a minimum puncta size = 0.3, sigma = 0.75, radius = 1, method = Phansalkar.

**Programming and Macros**

This program was developed and run on FIJI v1.54f using publicly available functions. The code and affiliated ReadMe files are available at https://github.com/heinohv/puncta_analysis .

The program was designed to identify and describe puncta using the following characteristics: puncta intensity, puncta width (FWHM), and density. Below, we briefly outline the functions of 6 macros included in the program. ***Macro 1* :** 1_files_to_max.ijm. The user selects the folder that contains the images for analysis, and an output folder. By running Macro 1, .lif files, z-stacks, and other image types will be opened and saved into the output folder as max projected .tiff images.

***Macros 2*** *:* 2_worm_to_VNC.ijm. Allows user to trace a linear path that will be output at a straightened image of the selected (in-focus) puncta. ( Figure 1A ).

***Macro 3* :** 3.0_quality_check.ijm is an optional step that allows the user to discard or crop images that they deem are low quality or blurry.

***Macro 3.5:*** 3.5_find_settings.ijm allows the user to test 4 different thresholding settings with their data set ( Figure 1B ):

1. Minimum accepted puncta size, which determines the lower bound for area a puncta must be.

2. A sigma value, which determines the strength of the applied gaussian blur

3. A radius, which determines the region size auto thresholding will use for computation.

4. The method, which will pick different thresholding equations.

***Macro 4:*** 4_measure_puncta.ijm Uses the settings they determined in the previous step (Macro 3.5) for analysis of all images.

The macro will open one image at a time, and use the settings selected to select each puncta as a region of interest (ROI). Each ROI will have its width recorded (ROI width), its brightest spot recorded (ROI max intensity). The region of interest will have its intensity plotted from the leftmost side to the rightmost side. FIJI will fit a gaussian model to this plot. The width of the curve at half of the maximum value is calculated and recorded as full width half maximum.

Rarely, this curve will be misfit, giving an unrealistic value. This is caused when a puncta has a pattern ascending strongly to one side, and a sudden cut-off. If the full width half maximum value is greater than the width of the region of interest, a 1 will be recorded in a column named “discard” allowing the user to either filter out those values, or replace those values with the ROI width.

***Macro 5:*** *“* 5_measure_puncta_density.ijm”. Macro 5 calculates the density of puncta by dividing the quantity of regions of interest, by the length of the straightened image. The calculations are output as a table in ImageJ.

## Reagents

**Table:** 

**Strain name**	**Genotype**	**Available from the CGC?**
**FJ354**	*nuIs24 (glr-1::gfp)* IV	**no**
**KP3814**	*nuIs152 (Punc-129::GFP::SNB-1)*	**yes**
